# Exploring the lived experiences of pregnant women and community health care providers during the pandemic of COVID-19 in Bangladesh through a phenomenological analysis

**DOI:** 10.1186/s12884-021-04284-5

**Published:** 2021-12-05

**Authors:** Sadika Akhter, Feroza Akhter Kumkum, Farzana Bashar, Aminur Rahman

**Affiliations:** International Centre for Diarrhoeal Disease Research, 68, Shahid Tajuddin Ahmed Sarani, Mohakhali, Dhaka, 1212 Bangladesh

## Abstract

**Background:**

Like many countries, the government of Bangladesh also imposed stay-at-home orders to restrict the spread of severe acute respiratory syndrome coronavirus-2 (COVID-19) in March, 2020. Epidemiological studies were undertaken to estimate the early possible unforeseen effects on maternal mortality due to the disruption of services during the lockdown. Little is known about the constraints faced by the pregnant women and community health workers in accessing and providing basic obstetric services during the pandemic in the country. This study was conducted to explore the lived experience of pregnant women and community health care providers from two southern districts of Bangladesh during the pandemic of COVID-19.

**Methods:**

The study participants were recruited through purposive sampling and non-structured in-depth interviews were conducted. Data was collected over the telephone from April to June, 2020. The data collected was analyzed through a phenomenological approach.

**Results:**

Our analysis shows that community health care providers are working under tremendous strains of work load, fear of getting infected and physical and mental fatigue in a widely disrupted health system. Despite the fear of getting infected, the health workers are reluctant to wear personal protective suits because of gender norms. Similarly, the lived experience of pregnant women shows that they are feeling helpless; the joyful event of pregnancy has suddenly turned into a constant fear and stress. They are living in a limbo of hope and despair with a belief that only God could save their lives.

**Conclusion:**

The results of the study present the vulnerability of pregnant women and health workers during the pandemic. It recognizes the challenges and constraints, emphasizing the crucial need for government and non-government organizations to improve maternal and newborn health services to protect the pregnant women and health workers as they face predicted waves of the pandemic in the future.

## Background

The first COVID-19 case was identified in Bangladesh on March 8, 2020 and the first death was on 18^th^ March, 2020 [1]**.** As of October, 2021, the number of confirmed cases in Bangladesh was 1,569,539 with 27,868 deaths [2]**.** The poorly resourced health-care system of the country is facing enormous challenges to tackle the pandemic of COVID-19 [3, 4].

Like other countries, the Bangladesh government imposed stay-at-home (lockdown) orders to stop the transmission of the virus. These orders continued from 26^th^ March till the end of May 2020 [1, 5]**.** Further, the orders were not imposed with enough preparation. The Government of Bangladesh declared a nationwide “Stay at home” order for offices and business organizations from 26^th^ March and extended it up to 30th May, 2020 [1]**.** Educational institutes were closed from 16^th^ March, 2020 to 11^th^ September, 2021 for face-to-face learning activities and examinations [6]**.** In the initial period, the whole country was unprepared for such movement restriction: there was no information for people about the proper methods of maintaining social distancing (keeping three feet distance which was marked in public places) or using masks in appropriate ways [6]**.** The sudden movement restriction orders also resulted in loss of income for many people for which there was no appropriate mitigation planned [7]**.**

Around the world, despite the efforts to respond to the pandemic, it is obvious that the essential services of maternal health care in low-income and middle-income countries such as Bangladesh have been seriously disrupted [8]**.** The evidence suggests that in past epidemics, health systems in such countries have struggled to maintain routine maternal and newborn child health (MNCH) services and the utilization of services has decreased [9, 10]**.** A recent report estimates that globally, 116 million babies will be born during the pandemic of COVID-19 of which 2.4 million will be born in Bangladesh [11]**.** The data from the website of the Bangladesh Directorate General of Health Services (DGHS) indicates a significant decline in utilization of maternal and newborn child health services. Only half of the district hospitals (out of 63 districts) provided key emergency obstetric care [11]**.** In addition, in-facility delivery also decreased by 15-20 per cent in Bangladesh during the month of April-May in 2020 in comparison to the same months in 2019 [12]**.**

A recent systematic review found that pregnant women infected with COVID-19 are at increased risk of adverse pregnancy and birth outcomes [13]**.** In addition, these women have a greater chance of being affected by the interruption of routine essential services such as immunization, antenatal care and in-hospital delivery [14]**.** Limited data suggest that coverage of routine antenatal, postnatal care and immunization has decreased due to COVID-19 pandemic [15]**.**

The pandemic of COVID-19 and lockdowns have had distressing effects on people’s lives [16]**.** Statistical modeling has already been done to estimate the direct and indirect effects of COVID-19 on maternal and child mortality in low- or middle-income countries [17]**.** In addition, the traditional epidemiological quantitative research made the case numbers of deaths and infection of the COVID-19 visible. However little is known about how the pandemic and lockdowns have affected the life of pregnant women and the community health workers who care for them. Quantitative research is important of course, but traditional quantitative research cannot explain social realities, often overlooking the social implications of the pandemic [18]**.** They are not well able to explain the reasons for people’s behavior and their social interactions during the time of health emergencies [19]**.** Qualitative methods are well established given their open-ended nature and focus not just on “what” is happening but also on an exploration of “why” and “how” [19]**.** Our objective was to understand the experience and challenges of the pregnant women and community health care providers where traditional quantitative methods might not be able to explore; hence a qualitative method was employed as the most suitable option.

To our knowledge, no qualitative studies have been published on the lived experience and the everyday struggle of pregnant women and field level health care providers during the pandemic in Bangladesh. To support them effectively, it is necessary to gain insights into their lived experience. This study aimed to identify the challenges from the perspective of the pregnant women and community health care providers and the probable outcomes of these challenges.

## Materials and methods

### The study setting, study participants and data collection

We conducted an exploratory qualitative study in the community setting by conducting key informant interviews (KIIs) and individual in-depth interviews (IDIs) in four rural sub-districts, namely Shyamnagar, Kaliganj, Dacope and Koyra. Shaymnagar and Kaliganj are located in the district of Satkhira while Dacop and Koyra are in the Khuna district. These districts are situated in the south-western part of Bangladesh in the division of Khulna. The population of these rural areas is generally poor, with farming and fishing as the primary sources of income. Data was collected over a three-month period between April and June.

**Fig. 1:**
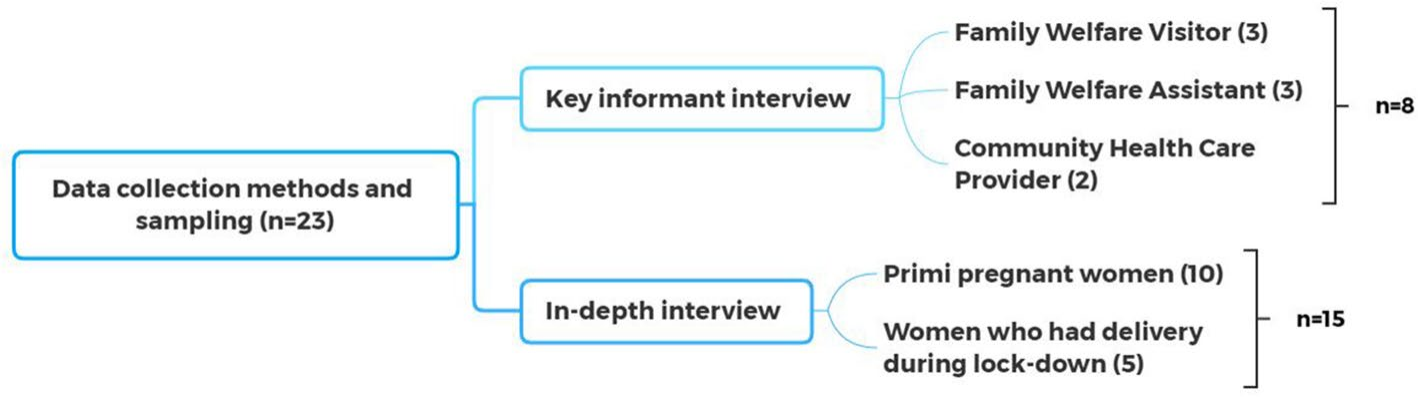
Data collection methods and sampling

A total of twenty-three interviews were conducted. The study participants were recruited through purposive sampling [20]**.** The sampling framework included interviewing the community health care providers, pregnant women and women who had delivered during the lockdown (Fig.1). Ten in-depth interviews (IDIs) were conducted with primi pregnant women who missed the 3^rd^ and 4^th^ ante-natal care sessions due to the lockdown, and five in-depth interviews were conducted with the women who had their first birth during the lockdown. “Primi pregnant women” means women who are pregnant for the first time in their life. The study selected primi pregnant women, as literature suggests that, in Bangladesh 15-24 year old women are more inclined to take ANC from medically trained health care providers, while ANC utilization usually decreases among pregnant women who have two or more children [21]**.** In addition, adolescent pregnancy is still highly prevalent in the country [22]**.** According to recent BDHS data, the median age of first marriage among women ranges from 15.5 years to 17 years. Demographic and health survey data also revealed that, among women aged 20-49, the median age at first birth was 18.6 years, and 66% of them gave birth before the age of 20 [23]**.**

Key informant interviewing is a critical component in qualitative research [24]**.** Key informants are regarded as experts in the area being researched and they impart important information to the researcher. The researcher encourages the informant to lead the discussion and elaborate on the topic. In this research, key informants were identified during the initial stages of the study data collection. The researchers conducted a total of eight interviews with the health care providers, which included three with family welfare assistants (FWA), three with family welfare visitors (FWV) and two with community health care providers (CHCP). During the interviews with the key informants, our focus was to elucidate their experience on how they provided health services to the pregnant women during the pandemic. They were identified as appropriate key informants because they were providing direct maternal and neonatal care health (MNCH) services and treatment at the community level to pregnant women. They are good sources of information as they are highly aware of what goes on, interact with a wide range of pregnant women from different backgrounds, and are able to represent the perspective of the general community. The in-depth interviews (IDIs) with pregnant women and key informant interviews (KIIs) with health care providers were conducted to deepen the data and to enable data triangulation. The data integration was done through data triangulation [25]**.**

Open-ended interview protocols were developed with initial themes to explore a) background information of the study participants, b) experiences of providing or receiving health services during lockdown c) health and safety issues for the health care providers d) challenges the participants faced during the pandemic in receiving or providing health services. The duration of the IDIs were forty-five minutes while the KIIs lasted forty minutes on average. All interviews were conducted with prior appointment according to the convenience of the study participants.

The first author leads the study with a PhD in anthropology and previous experience in qualitative research. She conducted the key informant and in-depth interviews with trained research investigators who have a background in sociology and public health. The telephone interviews were recorded and the interviewers took detailed notes during the interviews. Later the recordings were transcribed by the interviewers. In the process of gaining data from the study participants the researchers tried to be flexible, which allowed a more natural interaction between the researcher and the study participant [26].

As qualitative researchers, the authors are part of the research process [27]**.** The authors tried to be neutral in exploring the lived experience of the study participants. Nevertheless, the authors had little to no personal experience with the topic of the research and were unable to relate to it. In relation to this research, the authors examined their pre-understandings in several stages by discussion among themselves. First, the authors had discussions to ensure that many areas emerging from the literature were explored in regard to the research topic. Second, the interviews were transcribed verbatim and the authors reviewed the data from the interviews, concentrating on their pre-understandings specifically where differences were found. Third, the authors reviewed the data again to determine whether their pre-understandings had changed in light of the data they had reviewed. Finally, the data were analyzed with the authors pre-understandings [28]**.**

All interviews were conducted over the telephone and verbal consent was obtained before conducting each of the interviews. A sample of 23 interviews was conducted and the codes were identified by the 12^th^ interview and data were saturated by the 23^rd^ interview [25]**.**

Ethics approval for this research was received from the institutional review board of the International Centre for Diarrhoeal Disease Research, Bangladesh (icddr,b). Verbal informed consent was obtained from each of the respondents during the telephone interviews. All personal identification of the respondents was removed from the interview transcripts except their role, namely, health care provider or pregnant woman/woman who had delivered during the pandemic. Numbers were assigned instead of names {(e.g., CHCP1, FWV1, 2, etc. and FWA 1,2, pregnant woman (PW 1, 2) and woman who had delivery (DW 1, 2, 3)}.

### Data analysis

**Fig. 2:**
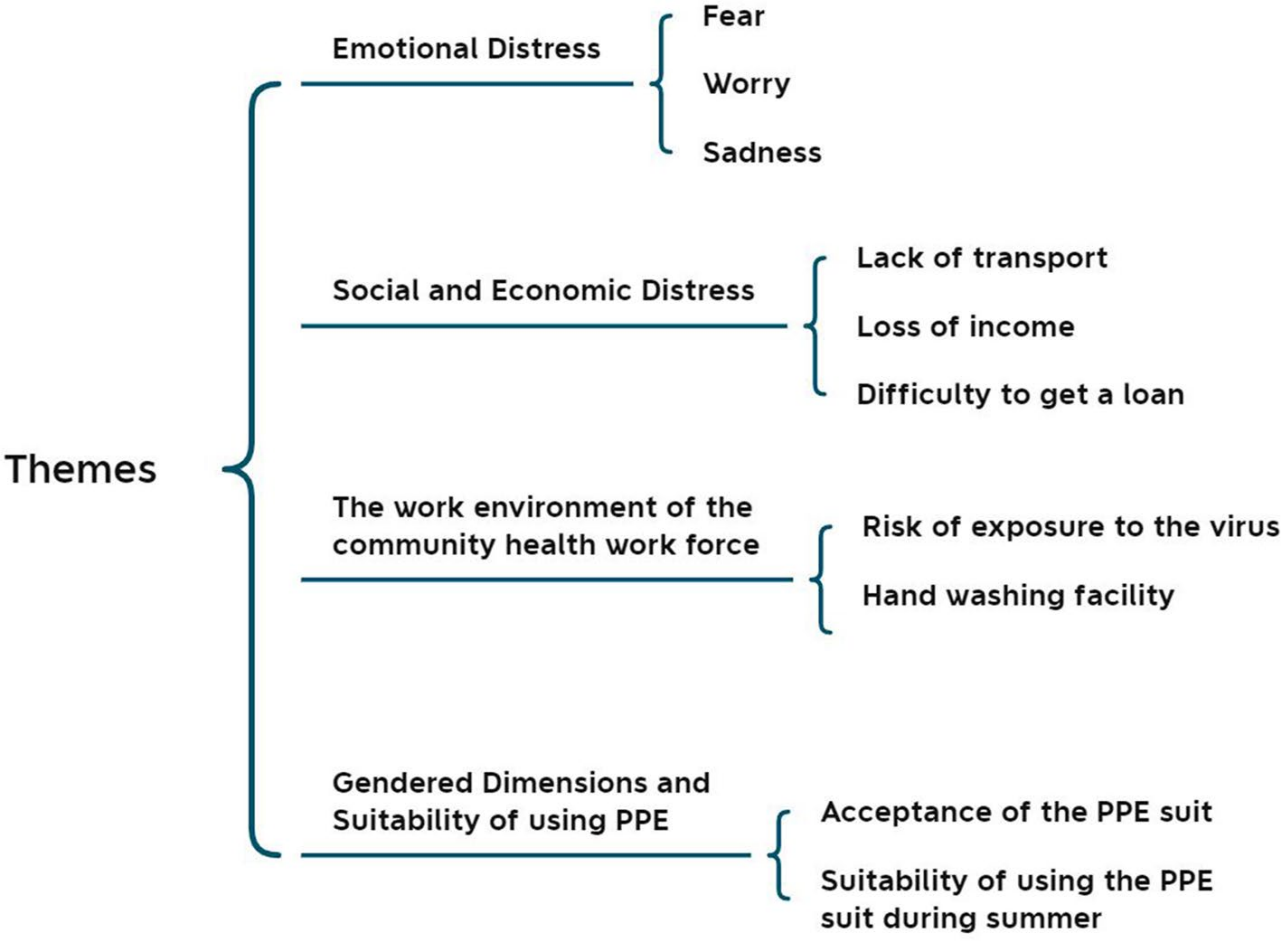
Themes of the study findings

Data analysis was conducted according to Colaizzi’s (1978) phenomenological approach, which helps to explain the lived experiences of the study phenomena across a homogenous population [26]**.** The first author (SA) was responsible for the comprehensive analysis of the interviews using Colaizzi’s method [16]**.** The data collection and analysis were conducted concurrently, including the repeated reading of interview transcripts to reach a complete understanding of the data gathered through KIIs and IDIs. Interviews were conducted by three of the authors (SA, FB and FAK)). At the end of each interview, the researchers compiled their interview notes. The interview notes were expanded with a partial narrative. The interviewers reviewed the narratives to identify emerging themes. In the subsequent interviews, questions were added about the emerging themes. The authors had debriefing sessions through Skype after the interviews. The interview notes were expanded after each of the interviews. Transcripts were checked and compared to the detailed notes to ensure consistency and accuracy. Fifteen codes were derived from the descriptive texts. Initial codes were then compared and discussed by two authors (SA and FAK) until they reached agreement on themes and sub-themes. These codes were clustered under four main themes: emotional distress, material deprivation, the work environment of health care providers, and the gendered nature of personal protective equipment (PPE). A total of ten subthemes emerged from the four main themes and those are presented in Fig. 2.

## Results

The socio-demographic information about the pregnant women is presented in Table 1. All of the pregnant women were 18-19 years old except two who were 20 and 21 years old. Ten participants had completed education up to primary school (grade 5), five participants were educated up to grade 10, three had received home education and two had completed high school. All of the women were housewives. The husbands were working as day laborers and farmers except one who is a school teacher.

**Table 1:** Background information of IDI participants

Type of respondent	N
Pregnant women	**10**
Women who had delivered	**5**
**Age (years)**	
18-19	**13**
20-21	**2**
**Marital Status**	
Married	**15**
**Number of children**	
1	**5**
0	**10**
**Religion**	
Muslim	**10**
Hindu	**5**
**Education**	
1-5	**10**
6-10	**3**
10^+^	**2**

## Theme I: Emotional distress

### Fear, worry and sadness

Several types of emotional distress were frequently stated in this study: fear, worry and sadness. The pregnant women became nervous due to the fear of getting infection from the corona virus. They were terrified as there was nowhere to go for treatment if they got sick from the corona virus during the pregnancy. The pregnant women were experiencing feelings of sadness as they were feeling that they would die if they got infected.

#### Fear

Interviews with the pregnant women revealed that they hesitated to seek care from health facilities for fear of exposure to the virus and they faced barriers to visit the health facility for ante-natal check-ups due to the lockdown-related mobility restrictions. Furthermore, they believed that the health facilities were the places where they would contract the virus. So they stated that they stopped visiting the health facility for fear that the ‘blood pressure machine’ and the ‘weighing scales’ would give them the corona virus.

One of the respondents (18 years old) in the last weeks of her pregnancy said, *“This is my first pregnancy. It is a time to be joyful. But the virus (COVID-19) has turned my happiness into anxiety as many people are getting infected from the virus. If I go to the health facility, the doctor would check my blood pressure and weight. I would get sick form this corona virus from these machines. I stopped visiting health facilities I didn’t go for my scheduled ANC visit.”* (PW1)

#### Worry

Every pregnant woman stated that she was worried about the life of her child and herself. If the labor pain started, where would she go and how would she reach the hospital? These were a constant worry for all of them.

*“I and my husband are really worried about my delivery. I always think that if the pain starts at night and if complications arise, how will I reach the hospital? We decided to have the delivery at home even if I die. If I have any complication, then how I will reach the hospital I don’t know. It constantly worries me.”* (PW2)

*“My delivery is due within next two weeks. I cannot sleep at night due to the tension about how I will go to the hospital. I heard that doctors are not available at the hospital. If I reach the hospital, if there is no doctor then how will I and my child survive?”* (PW3)

#### Sadness

The study participants indicated that the most joyful event of their life suddenly had turned into sadness. The main causes of sadness are fear of death, possible exposure to the virus, and financial burden.

One respondent who is 18 years old said, *“I feel sad always. If I get infected due to this virus, I will die. I shared it [this thought] with my husband. My husband said that he wants the tree [tree means here the pregnant women and fruit means here the child] to survive first not the fruit. If the tree survives then he will have an opportunity to have the fruit again. If the tree does not survive, what will he do with the fruit only?”* (PW4)

To deal with these emotional distresses, all respondents stated that they started to pray more when they heard about the virus. Religious beliefs were powerful among the respondents as they prayed to God/the gods to save them from the virus during the pregnancy. All respondents confirmed that they pray to God/the gods and always recite spiritual verses from their holy books to save themselves and their children from the virus. Prayer helps them to cope with fear, worry and sadness.

In this regard one woman who is 18 years old said, “*I cannot sleep at night due to the fear of the virus and death. I always pray to god to save the life of us. We are poor people: if God doesn’t save us, who will save us? Allah (God) has sent the virus to the world and only he can save us from it.”* (PW5)

## Theme II: Social and Economic Distress

This theme focused on the different types of material deprivation after the lockdown was imposed. The subtheme “lack of transport” focuses on the experience of distress related to suspension of transport due to the lockdown. In their community, there is only motor cycle to take people to the health centre for ante-natal check-ups and having delivery. The subtheme “loss of income” focuses on the distress of their husbands feeling trapped at home unable to earn an income during the lockdown. The subtheme “difficulty to get a loan” focused on the concerns of the pregnant women due to their husbands’ loss of income. They usually get financial support from their relatives by borrowing money but it may not be accessible during the time of the lockdown as everyone is in the same predicament.

### Lack of transport

Our data revealed that none of the pregnant women who were on their third trimester of pregnancy during lock-down could go to the health facility for ANC visits because of the non-availability of transport due to the pandemic-related lock-down and restrictions on mobility.

*In this regard one pregnant woman said, “The health facility where I go to take ANC visit is one-hour ride by motor cycle from my home. This is the only way to get there. My husband and mother-in-law suggested me not to visit the health facility as vehicle is not available. I called to the doctor (FWA) and she also suggested me not to go the health facility. She gave me advice over the phone.”* (PW6)

*Another woman who had her delivery during lockdown said, “I had my labor pain more than one day. My husband called a health worker over the phone to do the delivery at home. The health worker came to my house. After examining me, she suggested taking me to the hospital as the position of the baby was breech. But the government hospital is far from my house. I need to walk mostly over a muddy road, to reach the main road. My husband managed a motor cycle but I couldn’t sit on motor cycle with my labor pain.” She further added, “I felt like I would die on my way to the hospital, if I sit on the motor cycle with severe labor pains. But in the end, I with the grace of the Almighty safely delivered a girl at home.”* (DW2)

### Loss of income

All of the pregnant women reported financial constraints as one of the major barriers to having a delivery in the hospital. They further stated that due to the lock-down, their husbands were mostly at home without work and income. They lost their income and now the cost of delivery is a matter of constant anxiety if the delivery takes place at the hospital. All the pregnant women reported that they wanted to have their delivery at home to reduce the financial burden on their husbands.

In this regard one respondent said, *“My husband is at home. He now cannot go for work due to the lock-down. He has lost his income. If he could continue his work and earning, then we would plan to go to the hospital for delivery. Now I just sit and pray to God to help me to have delivery at home and also to save the life of my child and mine too.”* (PW7)

### Difficulty to get a loan

The respondents indicated that their husbands work as day laborers. They cannot go out for work due to the lock-down and thus are unable to continue their income. The respondents indicated that there is no place to go to borrow money because everybody is in trouble in the village. Some participants reported that they would sell their livestock and gold jewelry and would take a loan with interest if they have to go to the hospital for delivery.

In this regard one pregnant woman said, *“I have some gold ornaments which I got as gifts when I got married. My husband now cannot go out for work. If I need to go to the hospital for delivery, we will sell our ornaments to manage money to bear the cost of the delivery.”* (PW12)

Another woman (19 years old) who had already delivered explained,

*“I had my delivery in the private clinic. We didn’t go to the government hospital as we heard that doctors are not available at the hospital due to the corona virus. We went to the clinic as one of our relatives works there. My husband works as a school teacher in a private school. The schools are closed due to lock-down and my husband is not getting his salary. We borrowed money from one of our relatives but I don’t know how we will pay our relative back if my husband doesn’t get his salary.”* (DW3)

## Theme III: The work environment of the community health work force

This theme focused on the experiences of the community health workers during the pandemic of COVID-19. Two subthemes emerged: risk of exposure to the virus and lack of hand washing facility. Nevertheless, the community health workers continued to provide essential health services under challenging conditions including limited opportunity to wash hands and inadequate support. Further, they lack masks, gloves, and hand sanitizer/ soap and these shortages are likely to aggravate their own and the communities’ chances of contracting COVID-19.

### Risk of exposure to the virus

The subtheme-risk of exposure to the virus Interviews with the community health care providers found that the health workers are at risk of disease exposure. They are providing health services by risking their health and their own lives. The respondents reported that the government provided masks, gloves, PPE gowns and other essential supplies but the number of the PPE kits is very insignificant. The respondents also mentioned that they are currently not only facing a shortage of PPE, but also there are concerns about shortage of other supplies such as medicines, sanitizing items and the sexual and reproductive health commodities which may hamper their ability to meet the needs of women and girls with quality care. Though respondents reported that their clinic has some medicines, due to the mobility restrictions they expected to run out of stock and would face problems in refilling their stock.

One health care provider said, *“I received two surgical masks, one gown and a few sets of gloves which is not enough to provide health service to the patients.”* (FWV1)

### Hand washing facility

The health care providers reported that there is no supply of water for hand washing in the health facility. They carry water from a nearby pond. When patients come to the health facility, they cannot provide water for them to wash hands. They maintained a distance when seeing the patients during this pandemic but the patients were not happy about this.

One health care provider described her concerns regarding their risk of working during the pandemic of COVID-19, *“Even in this pandemic we are providing health services despite limited facility for washing hands. We are visiting households; otherwise there will be a rise in maternal deaths. We are doing deliveries at home when the pregnant women call us during their labor. Nobody is there to look after our health except God.”* (FWV3)

The KIIs reported that it is difficult to maintain hand washing at the health facility regularly not only because of the shortage of water but also because of a shortage of soaps and hand sanitizer. They further reported that their health facilities do not have an isolation area or separate consultation room for suspected cases. The community health care providers expressed their frustration that they are risking their lives at work without enough personal protective measures.

Another health care provider presented following clarification for not encouraging pregnant women to visit health center in person for ANC check-up, “*Most of the pregnant women of our village who need to have an ANC check-up are not visiting health facilities. We also do not encourage the pregnant women to visit the health facility. We encourage them to call us over the phone. If they call us, we request them to stay home and we give health advice over the phone.”* (CHCP1)

## Theme IV: The gendered dimensions and suitability of using PPE

This theme focuses on the experience of the community health workers related to the suitability of using PPE from gender perspective as well as the appropriateness of using it during the time of summer to provide health service at the community level. The female community health workers refused to wear PPE to work because PPEs are designed for men, it is not comfortable for female health workers and especially not culturally appropriate as community members tease them if they wear PPE to work.

### Acceptance of the PPE suit

All of the health care providers interviewed are women. They all reported that they do not wear the PPE gown, which is designed for men and is shaped like trousers. They wear a *sari*, which covers the legs to the ankles. They wear local dress which is known as *“sari”,* a dress comprising of a length of cloth wrapped around the body, traditionally worn by women. Wearing a PPE gown with a sari is impossible. If they want to wear PPE, they need to wear another type of local dress known as “Salowar and Kamiz” consisting of loose trousers and a long top, but they do not have any. They further mentioned that when they wear the PPE gown suit they look like a man. The villagers stare at them, tease them and they do not feel comfortable to go out wearing a PPE suit.

A health care provider described her experience about her reluctance to wear PPE: *“I wore the PPE gown and the villagers laughed at me. They teased me by calling me “Kangaroo”. I also felt that I look like a man. This suit is not suitable for a female to wear. I do not wear the PPE suit.”* (FWA1)

### Suitability of using the PPE suit during summer

The family welfare visitors added that when they visit from home to home to provide health services they do not wear PPE because they get sick from excessive sweating when they wear the gown during this hot and humid weather.

One health care provider provided following explanation about the use of PPE, “*I received one gown. I visit households in the village on foot and it is not possible to visit households in this hot summer while wearing a gown. One day I wore it and I got seriously sick due to the excessive sweating for the hot summer. I also carry almost 10-kilograms [of equipment] with me including four big register books, one carrier of vaccine and some other equipment with me. It is not possible to visits houses while wearing PPE gown. I will then die from suffocation due to the hot summer.”* (CHCP2)

## Discussion

Though the maternal mortality rate has declined in Bangladesh, which indicates notable progress, it is still one of the highest rates in the world [19] Studies have found that many factors contribute to the high rate of maternal mortality [27]**.** These factors include poverty, limited access to health services, gender inequality and lack of skilled health care providers [28]**.** The safe motherhood initiative and seeking recognized antenatal care (ANC) are pillars ensuring safe pregnancy and delivery [29]. The lives of pregnant women will be saved by ensuring this essential service during the time of pregnancy and childbirth [29]. Studies have revealed that insufficient provision of ANC is one of the reason for maternal morbidity and mortality in developing countries [30]. Similar to other studies, our study recognized a lack of access to the antenatal care due to the lockdown without enough preparation for COVID-19 placed women in emotional distress [30, 31]**.**

The lived experiences of the pregnant women and community level health workers give a comprehensive view of the challenges they are facing during the pandemic. The community health workers play a vital role at the community level by providing essential maternal and child health service for the pregnant women. But their responses reveal the existence of several challenges from the different standpoints of the pandemic. The lived experience of the pregnant women and health care providers also indicates how powerless the women and community health workers feel. Our finding is consistent with the other studies [32, 33]**.**

The narratives of the study participants identified several challenges especially mental health stress. The health workers pinpoint work environment factors, such as limited facilities for hand washing and difficulties with other protective equipment (lack of gloves etc., inappropriate PPE, and heat) and their own and the communities’ perceptions about wearing the PPE suit. Despite the stress, the health care providers continue to make great efforts to provide the health services, carrying out their responsibilities, focusing on their duties, and showing professional commitment. A previous study also made similar findings [33]**.**

The study identifies issues of gendered PPE that lead health workers to abandon PPE while working. Considering the predictability of a second wave of the COVID-19 pandemic, communities and health professionals can design locally appropriate PPE to ensure that appropriate PPE is worn.

Pregnant women identify the period of their pregnancy as worrying and unpleasant due to stress, anxiety and depression. Further, their lived experience during lock-down indicates that their daily life is severely affected due to the stress. The lived experience of the study respondents demonstrates their resilience and spirit to survive during the pandemic. They used prayer and strength of will to deal with the stress, because they knew that they did not have enough resources to tackle the pandemic. However, the community level health-care providers of this study also expressed unhappiness and powerlessness about not being able to provide health services to relieve the suffering of the pregnant women during the pandemic [34]**.**

Access to emergency health services was also severely hampered due to the lack of available transport, shortage of PPEs along with trained human resource, and absence of COVID-19 screening facilities as well as proper guidelines for admitting non-COVID-19 patients in different hospitals [35]. Further, at the beginning of the pandemic in the March, 2020, most of the government and non-government hospitals didn’t have triage (separating COVID-19 patients from non-COVID-19 patients), handwashing and other hygiene facilities and supply of personal protection equipment [36]. As a result, the pregnant women and health care providers are living on the edge of fear, pressure and despair. The country had to prioritize controlling the spread of the COVID-19, but this effort has significantly disrupted the maternal and newborn child health services, particularly ante-natal care, safe delivery and post-natal care, and has proved that the one-size solution does not fit for all.

The community health workers are providing essential health services in overcrowded conditions, without access to water to wash hands, and working in a sector which leaves them in a position to be susceptible to infection. Still, despite the stress, challenges and limited resources the health workers are providing this valuable service to their communities at the risk of their lives [35]. This study presents some key recommendations in Table 2.

**Table 2:** Recommendations for action

**1. Work environment:** Primarily, the results suggest that action should be taken by key stakeholders to create a supportive work environment with sufficient supply of PPEs and appropriate infrastructural setting for health hygiene and hand washing practice to prevent infection of COVID-19.	
**2. Gender appropriate PPEs:** Gender appropriate PPEs should be developed for the community health workers so that they can continue their work at the community level with appropriate personal protective equipment.	
**3. Social phenomena:** A strong community based awareness-raising intervention targeting all community members is essential to enable pregnant women to continue to access the essential existing maternal and neonatal care health services while following proper COVID-19 preventive measures.	

### Strengths and limitations

Historically interviews by telephone have been conducted to collect qualitative data [37–39]. During the time of COVID-19 interviewing by this technology instead of face to face has increased to reduce infection of COVID-19. The authors of this study collected data through the use of telephones [40]**.**

The study findings should be viewed within the limitations of the study. Face to face interviews with direct interaction between researchers and study participants to collect data are important to develop rapport between researcher and study participants. Direct interaction during data collection helps to understand the environment – where and how the study participants live and work. Since our data was collected over the phone, this may have influenced the rapport building between study participants and researcher [41, 42]**.** To mitigate the limitations, the study participants included only women who are accessible by telephone and those who own a phone, and who are willing to respond to questions over the phone. In addition, telephone interviewing is suitable while questions are open ended because study participants provide responses based on their memory. The challenges of developing relationships were mitigated by conducting briefing sessions for the researchers over phone prior to conducting actual interviews. Despite the limitation, this study identifies a number of realities that hinder the provision of effective maternal and child health services and hinder women utilizing these services.

### Implications

The policy makers, health program managers and development practitioners can use the findings of the study to prepare the health facilities to face the predicted multiple waves of the pandemic to provide essential health services to pregnant women, who are at higher risk. The preparations are generally not very expensive; however, they need to take into account the need for facilities that suit the realities of life in Bangladesh, especially the realities of gender in this society.

## Conclusion

The pregnant women and health workers are facing new challenges to receive and provide health services. To our knowledge, no qualitative studies have explored the lived experience of pregnant women and front line community health workers during the pandemic. The findings of this study will help policy makers and program designers to support them more effectively in the future in order to protect these vulnerable groups from the virus.

In conclusion, the study findings highlight the limitations of the health system, and some of the socio-cultural and economic factors that hinder the pregnant women seeking health services from the health facilities. The study has also indicated the stresses upon health workers in the maternal and child health system, especially the fear of infection and the struggle with inadequate supplies and equipment.

## Data Availability

The original data are not publicly available to ensure the study participants’ anonymity and privacy, but are available from the corresponding author on reasonable request.

## Abbreviations

FGD: Focus Group Discussion
IDI: In-depth Interview
KII: Key Informant Interview
